# Collectanea

**Published:** 1827-04

**Authors:** 


### COLLECTANEA.

Floriferis ut apes in saltibus omnia libant,

Omnia nos, itidem, depascimur aurea dicta.

### PHYSIOLOGY.

### *Remarkable Case of Triple Dentition.*

Elizabeth, wife of Dominique Morelli, healthy, and always having enjoyed good health, with the exception of toothaches, (for which she was, each time she had them, obliged to lose blood, sometimes repeatedly.) She was mother of four infants. In one attack of toothache, towards the middle of the month of March, 1821, after having suffered very much from the two last molares of the left side; she had them drawn. However, towards the end of October of the same year, extremely sharp pains preceded the cutting of two new teeth, which replaced those removed. In January, 1826, these new teeth becoming loose, and causing much pain, were removed also: they were white and beautiful, without any appearance of caries.

July 16th, M. Aimonino, who relates the case, was consulted by Madame Morelli, who complained of intolerable pains, and had an inflammatory toothache. Antiphlogistics and depletions were tried, without success; and, on the 18th, the patient discovered that a third supply of these two teeth was about to make its appearance; accordingly they soon protruded through the gums, when the pains ceased.

(*Repert. Med. Chirur. &c. de Torino.*)

### PATHOLOGY.

*Metastasis of the Milk.*—We are much surprised to find M. Graefe, of Berlin, report the following as a case of metastasis of the milk.

A young woman, the wife of a miller in Saxony, had been lately confined, and was nursing her babe. Eight days after her confinement, in consequence of some accident occurring to the mill, she was so frightened that the secretion of milk ceased. A violent intermittent fever supervened, which became a tertian. In the course of the fever, the patient had œdema of her legs, which daily increased. Three weeks after, this œdema became general. As the ascites was considerable, and means were inefficacious, M. Graefe was called in to puncture the abdomen. The puncturing gave issue to a quantity of fluid like whey, smelling sour, and which, submitted to boiling with diluted sulphuric acid, coagulated, and gave a perfectly cheesy substance. Diuretics were prescribed; the solution of Acetate of Potass, Digitalis, Squills, Juniper, &c.; but in vain: it was necessary to repeat the puncturing. This time the fluid evacuated was glutinous, of a greenish yellow, and contained no cheesy matter. After this the patient gradually got better under the use of diuretics and tonics.

(Hufeland's *Journal.*)

### PRACTICAL MEDICINE.

*Mercurial Frictions in Puerperal Peritonitis.*—M. Velpeau, in a Memoir published in the Revue Medicale, after deploring the inefficacy of our remedial treatment of puerperal peritonitis, proposes, as a means of cure, mercurial frictions on the belly. He orders two drachms of mercurial ointment to be rubbed in on the surface of the abdomen, and repeated every two or three hours. Much depends on the regularity with which this is done. After two or three frictions, should the symptoms not be ameliorating, he has the belly anointed with oil, and washed with soap and water, and the frictions recommenced. One would at first expect that the acute pain felt by patients in the abdomen would prevent the use of frictions: he says, that pain is not in the skin of the abdomen, but in the inner side of the muscles of the parietes; and therefore, that very gentle frictions with the hand of a person tenderly interested in the fate of the patient, does not increase the suffering, and, after being done once or twice, the pain is so much relieved that more freedom can be used. "It is wonderful (he says) how much more of the mercurial unguent may be made to pass into the general mass of fluids in this way, than when frictions on the thighs are used." With regard to the length to which these frictions may be carried, he thinks they should be continued some time after marks of salivation have shown themselves. It is necessary that the patient be kept in a temperature considerably elevated, and carefully guarded against cold currents of air. As to the mercury, it is evident, he says, that it acts, first, in modifying the nature of the fluids, and, by consequence, the state of the inflamed surfaces. He draws the following conclusions:

1st. That puerperal peritonitis, when well established, and left to itself, is almost always fatal. 2d. That it is still to be proved that, in this state, sanguineous depletions are a sufficient remedy. 3d. That it is incontestable, according to the observations of Hamilton, Gordon, and M. Vandenzende, that, by the aid of calomel in large doses, many women affected with this disease have been saved. 4th. That mercurial frictions made on the abdomen, frequently repeated, promise great success, and deserve to attract the attention of practitioners. 5th. That, by the aid of these, patients, who have had one foot in the grave, have been saved; and that, therefore, they ought to be used in any stage of the disease, however late. 6th. That they can be continued with great safety till the mouth shows itself to be affected. 7th. That it will be useful to add to their use that of baths, calomel, and an elevated temperature. 8th. That the facts observed by him, although not sufficiently numerous to produce conviction, yet may encourage practitioners to renew the trials. 9th. In fine, that mercurial preparations have the property of sometimes curing puerperal peritonitis, when well marked, but that, to discover the best mode of administering them, we must wait for farther experience.

*On the Impropriety of applying Ice to the Head in Cerebral Inflammations.*—At a late meeting of the Academie Royale de Medecine, M. Costa read a Memoir on the Treatment of Cerebral Inflammations, in which he states his dissent to the employment of ice to the head in cases of Arachnitis and Encephalitis. "Would we found this treatment (says he,) on the idea that the inflammation of organs contained within the brain is of a peculiar nature? But MM. Tomasin and Broussais have sufficiently proved that inflammation, wherever situated, and whatsoever its causes, is always the same. Now, then, if cerebral inflammations are the same as phlegmasiæ of other organs, why treat them differently from the others? Can we expect to oppose the flow of blood by the intensity of the cold?" The author thinks it produces quite a contrary effect, in constringing the vessels of the scalp, and forcing the blood in these vessels to flow back on the brain. Discarding the application of ice and blisters on the head, he proposes another mode of treatment.

In idiopathic cerebral inflammations, he shaves the head, and covers it, in the course of the sagittal suture, and chiefly at its posterior extremity, with a great number of leeches ; he then covers it with emollient poultices, which he renews as necessary; and also uses some general bleedings, if required. If, on the contrary, the inflammation is sympathetic with gastro-enteritis, which is commonly the case, particularly with children, he directs his attention to the state of the intestinal tube, unless the encephalitis greatly predominates, and then he treats it as above.

In concluding his Memoir, he gives these reasons why he prefers the sinciput for the application of the leeches:—The first is, that inflammation of the arachnoid, or encephalitis, usually has its seat in the anterior regions of these organs. 2dly. In applying leeches to the sinciput, he disgorges more directly the inflamed parts, as he acts on the superior longitudinal sinus, or rather on the veins which discharge themselves into it. 3dly. Because there is a sympathy between the skin which covers the splanchnic cavities and this part.

---

### SURGERY.

*Method of avoiding Amputation of the Penis in Cases of Cancer.*—In the Report of the Clinique de la Pitié, by M. E. Margot, an account is given of a method that M. Lisfranc follows, to discover to what degree of depth cancer of the penis extends, and whether the diseased parts may not be completely removed without amputating the penis.

M. L., in his clinical lecture, in showing a patient with cancer of the penis, took the opportunity of saying that he would put in execution an idea that he had long entertained, and which had been suggested to him from minute examination of some amputated cancerous penes. " I have seen (says he,) penes enlarged to twice their natural size, and with all the symptoms of occult or ulcerated cancer: every thing announced that this frightful disease completely occupied the organ. They have been removed, and, on minute examination, the disease has been seen to be confined to the corpora cavernosa." How are we to know that such is the case? There is but one way: it consists in making, on the dorsum of the penis, parallel to the axis of that organ, an incision, which, beginning at the anterior part of the cancerous point, shall be continued to the posterior. The bistoury must be used with much caution and slowness, and, as the incision goes on, the parts must be cleaned with a sponge; and, the extent of the disease being discovered, a careful dissection of the diseased parts may save the penis. The only inconvenience resulting from this operation is the prolongation of the sufferings of the patient.—M. Lisfranc has twice put in practice this operation, and each time saved the member. (*Rev. Med.*)

### MISCELLANEOUS.

*Means of Restoring those apparently Drowned.*—A book, intitled "Essai Historique et Therapeutique sur les Asphyxes," has lately been published by M. Plisson, in which he suggests that insufflation by the mouth of another person is better than by any machine. He says, the air expired still contains eighteen per cent. of oxygen; it is warm, and impregnated with the pulmonary perspiration which accompanies it into the lungs, softening and rendering the air less irritating than the colder surrounding atmosphere. The person who is to insufflate ought previously to make two or three deep expirations and inspirations, so as completely to renew the air contained in the lungs, before introducing it into the lung of the drowned person.—Next to insufflation, M. Plisson considers the introduction of tobacco smoke, in glyster, as the most efficacious means to be employed. He says, "But of all glysters, that with the fumes of tobacco has been most praised, and, what is better than all reasoning, is this, that a great number of persons who have been drowned have owed their restoration to this alone, aided by slight frictions, insignificant in themselves. I think, then, (he says,) that those who blame this measure act very wrong; and perhaps they would not so hastily have condemned it, if, laying aside all theory, they had confined themselves to practical observations."

It appears that, of 934 persons apparently drowned, who had been succoured at Paris, from 1772 to 1778, 830 were restored; and it was during this period that, by the care of M. Pia, means for giving these glysters (*boites fumigatoires*) were established: it is well known that this great philanthropist, in the instructions which he communicated, always recommended their use as the principal restorative means.

